# Extrapair Paternity and Maternity in the Three-Toed Woodpecker, *Picoides tridactylus*: Insights from Microsatellite-Based Parentage Analysis

**DOI:** 10.1371/journal.pone.0007895

**Published:** 2009-11-18

**Authors:** Meng-Hua Li, Kaisa Välimäki, Markus Piha, Timo Pakkala, Juha Merilä

**Affiliations:** 1 Ecological Genetics Research Unit, Department of Biological and Environmental Sciences, University of Helsinki, Helsinki, Finland; 2 Finnish Museum of Natural History, University of Helsinki, Helsinki, Finland; University of Oxford, United Kingdom

## Abstract

Molecular techniques have revealed that avian mating systems are more diverse and complex than previously thought. We used microsatellite markers to determine genetic parentage, the prevalence of extrapair paternity and quasi-parasitism (*i.e.* situations where a male's extrapair mate lay in his nest) in a socially monogamous population of three-toed woodpeckers (*Picoides tridactylus*) in southern Finland. A total of 129 adults and nestlings, representing 5–9 families annually from 2004–2007, were genotyped at up to ten microsatellite loci. The results of genetic assignment tests confirmed that monogamous parentage characterized the majority (84.6%, 22/26) of broods, and that most (93.8%, 75/80) nestlings were the offspring of their social parents. Two of 80 nestlings (2.5%) in two of 26 broods (7.7%) were sired by extrapair males and quasi-parasitism occurred in 3.8% (3/80) of nestlings and 7.7% (2/26) of broods. Hence, the levels of extrapair parentage were low, possibly because both genetic polygyny and polyandry are constrained by the high paternal effort required for parental care. The co-occurrence of low levels of extrapair paternity and quasi-parasitism are discussed in light of ecological and behavioural factors characterizing the species biology.

## Introduction

Extra-pair fertilizations, which can result from females engaging in copulations with extra-pair males (extrapair paternity; EPP), or from males copulating with extra-pair females that lay eggs in the male's nest (extra-pair maternity; EPM or quasi-parasitism, QP) [1], is known from approximately 90% of the avian species (see [2]). EPPs are known to be common in passerines and less so in non-passerines [3]. Despite its ubiquity across avian species, the prevalence of EPP varies considerably within and among species (see [2]). In contrast to EPP, QP is rare and has been described in only a few bird species. However, a close examination of these studies revealed that unequivocal evidence for QP is slim due to possibility of rapid mate-switching and/or insufficient molecular work [4]. Hence, it remains unclear whether QP is generally rare, or whether its apparent scarcity reflects the difficulty of identifying it when occurring.

Genetic parentage studies have been conducted only in four out of more than 200 woodpecker species (see [5]). One of these is the three-toed woodpecker (*Picoides tridactylus*) which is typically socially monogamous [6], although occasional cases of simultaneous social polyandry have been recorded [5], [7]. However, more accurate parentage analysis tools for the three-toed woodpeckers would be needed to address questions relating to the genetic benefits of mate choice, inbreeding avoidance and the actual breeding system in this species (cf. [8]). Likewise, additional data from non-passerine birds will be also useful in understanding the evolutionary significance and life-history correlates of promiscuity in birds. In comparison with the multilocus DNA fingerprinting analyses previously conducted in three-toed woodpeckers [5], application of high-resolution microsatellites would represent a more efficient and straightforward technique for parentage assignment and kinship analyses [9].

The main purpose of the present study was to estimate the prevalence of EPP and QP in the three-toed woodpecker. Since the males of this species allocate significantly more time to territory defence, cavity excavation and feeding of the young than females [5], we predicted that this should result in low frequency of EPP and possible occurrence of QP. The study was conducted in a population breeding in southern Finland, which has been studied since the late 1980s and has been a subject to a five-year (2003–2007) intensive population study (e.g. [10], [11]). To this end, we applied a set of 10 polymorphic microsatellite loci developed for the species [12]. In addition, given the statistical limitations facing most parentage studies (e.g. [13]), we further applied an approach which was implemented in program CERVUS and has been proven to be with high confidence in parentage assignment in an open mating system [14].

## Methods

### Ethics Statement

The methods were approved by the institution that coordinates ringing activity in Finland (Finnish Museum of Natural History), based on the regulation by the Ministry of the Environment (No. 17/5713/2002).

### Study Species

The three-toed woodpecker (*Picoides tridactylus*) is a cavity nesting habitat specialist inhabiting coniferous taiga forests in the north, and high elevation alpine coniferous forest at the southern edge of the boreal zone [5]. They exhibit nest-site fidelity over years [7], mate guarding [15], long duration of cavity excavation, bi-parental care, and in particular, a very high degree of paternal care due to exclusive incubation/brooding at night by the males [5]. The average breeding density varies a lot depending on the incidence of fire accidents [16] and the quality of forest landscape, habitats and spatial scale considered, and has been estimated to vary between 0.1 and 1.5 territories/km^2^ in an intensive studied area of 150 km^2^ in southern Finland [10], [11].

### Study Site and Population

The study was conducted in the Evo area (*ca*. 61°11′N, 25°06′E) in southern Finland in an area of 150 km^2^. The study area has been described in more detail in [10], [11].

The territory numbers and nest sites of the three-toed woodpecker population inhabiting the Evo area have been studied since 1987 [11]. An intensive study on breeding biology, including individual marking of birds with colour rings, was started in 2004. During years 2004–2007 territories and nests were searched in the study area by using methods described in [11]. Ten to 25 nests with nestlings were found annually. For each nest possible, the adults were trapped and nestlings were pulled out from the nest cavities with a special tool (soft tongs) that is in general use in the woodpecker ringing projects in Finland. The adults were trapped using mist nets or a net designed for catching birds coming out from the nest cavity. Each bird was tagged with an individual combination of colour rings, measured, aged, sexed (adults only) using the morphological criteria given in [6], and body feather samples were collected from the birds. Two to five feathers were plucked with a pair of tweezers from the ventral body feather tracts in case any feathers did not fall away during the handling of the birds. No adverse effects on birds were observed during or after the catching, ringing, measuring and feather removal. Finally the nestlings were put back in the nest and adults were released. Due to characteristics of trees, cavities or sites, all found nests could not be sampled completely. In addition, some nests were found too late in the course of the breeding season therefore pulling the large nestlings out from the cavity was no longer safe for the individuals. Altogether 26 nests were sampled adequately to further analyses.

### DNA Extraction, Molecular Sexing and Microsatellite Genotyping

Genomic DNA was isolated from the body feather shafts using the Chelex-based extraction protocol (Bio-Rad, Helsinki, Finland) following the manufacturer's instructions. The sex of all samples was identified following a simple and universal method for molecular sexing of non-ratite birds using PCR amplification of the CHD1 gene as detailed in [17]. In those cases where birds were sexed on the basis of crown feather coloration (e.g. [6]) in the field, the results of field and molecular sex identification methods matched each other perfectly. A total of 10 polymorphic microsatellites developed for the three-toed woodpecker [12] were included in this investigation (Table 1). The PCR genotyping protocols are available from [12]. All genotypes were double checked independently by two persons.

**Table 1:** Summary statistics for the 10 microsatellite loci used in this study.

Locus	*n*	*N*_A_	*H*_O_	*H*_E_	*F*_IS_	*F*_Null_	*P*_(Ex1_)	*P*(_Ex2_)	*P*_H-W_
Ptri13	129	11	0.767	0.768	0.001	−0.002	0.372	0.549	0.1761
Ptri17	126	11	0.817	0.845	0.033	0.027	0.525	0.692	0.5625
Ptri20	129	3	0.101	0.097	−0.036	−0.016	0.005	0.049	1
Ptri22	129	11	0.829	0.806	−0.029	−0.013	0.448	0.623	0.2304
Ptri23	123	7	0.715	0.662	−0.081	−0.045	0.264	0.448	0.1574
Ptri24	129	7	0.69	0.655	−0.054	0.028	0.232	0.392	0.2489
Ptri30	127	15	0.827	0.864	0.044	0.020	0.57	0.728	0.0346
Ptri31	125	6	0.864	0.8	−0.081	−0.044	0.422	0.6	0.4087
Ptri36	129	11	0.783	0.844	0.073	0.036	0.519	0.687	0.1451
Ptri38	127	9	0.732	0.782	0.064	0.032	0.426	0.609	0.0493
Overall	127.3	8.7	0.713	0.712	0.0001	–	0.9935	0.9998	–

Number of birds screened (*n*), number of alleles (*N*_A_), observed heterozygosity (*H*_O_), expected heterozygosity (*H*_E_), Weir and Cockerham's (1984) within-population inbreeding coefficient (*F*_IS_), frequency of null alleles (*F*_Null_), exclusion probability of the locus for the first parent (*P*_Ex1_), exclusion probability of the locus for the second parent with the first assigned (*P*_Ex2_), and the exact probability for deviation from Hardy-Weinberg equilibrium (*P*_H–W_).

### Microsatellite Variation

Deviations from Hardy-Weinberg equilibrium (HWE) for each locus and from linkage equilibrium between all pairs of loci were tested with Fisher's exact tests based on the approach of [18] using GENEPOP version 3.4 [19] with 100 000 steps in the Markov chain (100 batches with 1000 iterations). Basic diversity indices, including the number of alleles, observed heterozygosity, Nei's [20] unbiased estimates of expected heterozygosity, within-population inbreeding coefficient (*F*_IS_; [21]), and frequency of null alleles were estimated at each locus as well as over all loci using GENEPOP. Standard exclusion probabilities for each locus and for the selected loci combined (Table 1) were estimated with the program CERVUS 3.0 [14].

The distribution of genotypes at the ten loci conformed to the expectation of HWE and all the locus pairs were in linkage equilibrium (Table 1; *P*>0.05; data not shown for the results of tests for linkage disequilibrium). The cumulative exclusion probabilities for the set of loci used in the parentage analysis were high: 0.9935 for the first parent and 0.9998 for the second parent (assuming the first parent was assigned correctly; Table 1).

### Parentage Analysis

**Figure 1:**
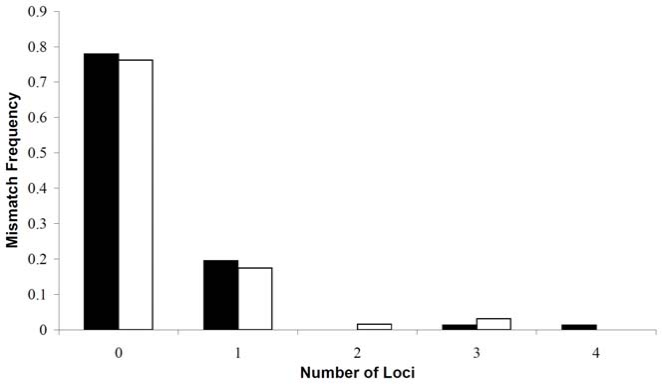
Histogram showing the frequency distribution of mismatches between each three-toed woodpecker nestling and its putative father (▪) and mother (□).

We first checked mismatch distributions between the putative parents and the nestlings. The fact that most offspring matched the putative mother or father exactly, or mismatched at a single locus, strongly suggests that most of the putative parents were true genetic parents. Of the cases where mismatches occurred, five mismatched by more than one repeat at two or more loci (Figure 1).

The parentage analysis of genetic data from the field-collected samples was further performed using the computer software CERVUS version 3.0 [14]. By using a likelihood-based approach described in [14], CERVUS calculates parentage inference likelihood ratios and generates a statistic, ΔLOD, defined as the difference in positive log likelihood ratios (LOD) between the top two candidate parents.

A total of 10,000 tests, which is thought to be sufficient in most cases [14], were used here. We define ‘candidate parents’ as adults of the population in a specific year. Both male and female three-toed woodpeckers were assumed to be capable of producing offspring in their second calendar year (one year old) and, therefore, surviving males or females from earlier cohorts were included as candidate parents for offspring born in later years. The number of candidate females was 10, 21, 34, and 42 and the number of candidate males was 13, 32, 48, and 59 for the 2004–2007 cohorts, respectively. The sampling of parents in the study area was not exhaustive, and it was estimated that *ca.* 25–40% of the adults were sampled depending on the year (M. Piha, personal observation). Thus, a sampling rate of 25% was used for the 2004 cohort and 40% for the 2005–2007cohorts. The proportion of successfully genotyped loci was on average 98.5% as estimated from the genetic data (see results). A typing error rate of 1.2% was incorporated into the simulation of maternity and paternity assignments. Assignments were carried out at a relaxed level of 80% and a strict level of 95%.

We assigned parentage under two scenarios of steps. (*i*) For complete families in which both putative parents were sampled (*N* = 46 nestlings in 14 broods), we first assigned maternity with unknown paternity using the program CERVUS. Once a female was assigned, we then attempted to assign paternity to either the putative father or a potential breeding male from the population with known maternity; otherwise, paternity assignments were implemented with unknown maternity. We included the putative parents when possible and all potential females/males in the population as possible candidates for maternity/paternity. (*ii*) For families which there was sample available for only the putative father (*N* = 34 nestlings in 12 broods) we again attempted to assign paternity using CERVUS with unknown maternity. There were no cases where a DNA sample was available for just the putative mother.

**Figure 2:**
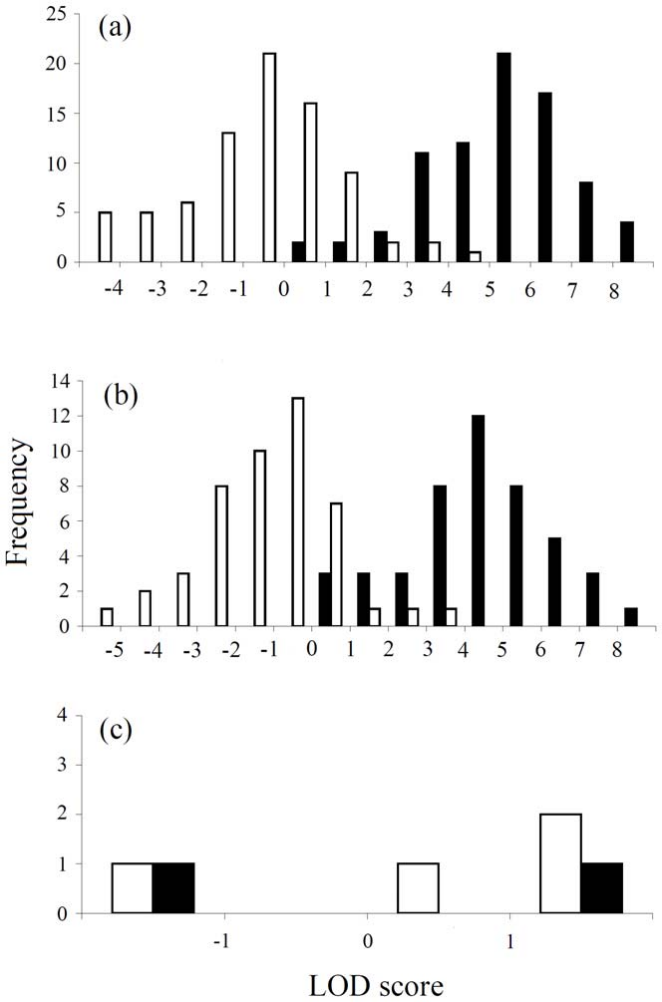
LOD score distributions from CERVUS analyses of parentage in three-toed woodpeckers. (a) LOD score for the candidate males that have been unequivocally assigned as fathers (▪) and for candidate males that were the second most likely candidate excluded (□) (*N* = 80); (b) LOD score for the candidate females that have been unequivocally assigned as mothers (▪) and for candidate females that were the second most likely candidate excluded (□) (*N* = 46); (c) LOD score of pair male with offspring assigned to extrapair father (▪) and pair female with offspring assigned to extrapair mother (□) (*N* = 5).

The distribution of the LOD scores of assigned and excluded parents is shown in Figure 2. The LOD scores of unequivocal within-pair offspring (*i.e.* assigned to putative parents) assigned on the basis of matching eight or more loci are all positive and the majority of them are greater than three (Figure 2a,b), but this is not true of the five extrapair offspring assigned with extrapair parentage (Figure 2c). The Δ criterion calculated for assignment of parentage was between 1.44 and 5.14 in different years for 95% confidence, and between 0 and 2.94 for 80% confidence where one parent was known (Table 2).

**Table 2:** Critical ΔLOD scores and actual and predicted success rate of ten microsatellite loci used to assign parentage.

	Maternity assignment				Paternity assignment			
	95% confidence		80% confidence		95% confidence		80% confidence	
Year	ΔLOD	Rate	ΔLOD	Rate	ΔLOD	Rate	ΔLOD	Rate
2004	1.79	55 (60)	0	100 (100)	1.53	89 (95)	0	100 (100)
2005	1.44	67 (75)	0	100 (100)	1.92	88 (94)	0	100 (100)
2006	2.01	57 (67)	0	100 (100)	2.66	87 (94)	0	100 (100)
2007	2.33	49 (53)	0	75 (100)	5.14	85 (90)	2.94	95 (100)

Calculations were performed across the samples, expressed as percentage of total number of individuals analysed (predicted success rates in parentheses).

## Results

Across the years, we assigned 56.5% (26/46) of offspring to the putative mother with a high degree of confidence (*P*>95%) in the 14 complete families. Moreover, the putative mothers were confirmed as the genetic mothers of offspring for the vast majority of cases (93.5%, 43/46) with >80% confidence (Table 3). Of the 14 broods, 12 with two or more chicks, all offspring were assigned to the same putative mother.

**Table 3:** Details of parentage assignment analysis using CERVUS, including the sampling year, the nest identity, the number of nestlings in the nest (*n*), the availability (+) and unavailability (−) of social fathers (♂) and social mothers (♀), the number of nestlings assigned to the social fathers and social mothers using CERVUS with 80% confidence, the rates of extra-pair maternity (QP) and extra-pair paternity (EPP).

		**			CERVUS			
Year	Nest identity	n	♂	♀	Maternity assigned to ♀	Paternity assigned to ♂	QP	EPP
2004	Evo13	4	+	+	4/4	4/4	−	−
	Evo14	2	+	−	−	2/2	−	−
	Evo15	4	+	+	4/4	4/4	−	−
	Evo16	3	+	+	3/3	3/3	−	−
	Evo19	3	+	+	3/3	3/3	−	−
	Evo20	3	+	−	3/3	3/3	−	−
	Evo21	4	+	+	4/4	4/4	−	−
	Evo22	2	+	−	−	2/2	−	−
	Evo23	3	+	+	3/3	3/3	−	−
2005	Evo24	4	+	+	2/4	4/4	2/4	
	Evo25	3	+	+	3/3	3/3	−	−
	Evo27	2	+	−	−	2/2	−	−
	Evo28	2	+	+	2/2	2/2	−	−
	Evo34	3	+	−	−	3/3	−	−
	Evo37	3	+	+	3/3	3/3	−	−
2006	Evo40	3	+	+	3/3	2/3	−	1/3
	Evo41	4	+	−	−	4/4	−	−
	Evo42	3	+	−	−	3/3	−	
	Evo44	3	+	+	3/3	3/3	−	−
	Evo45	2	+	−	−	2/2	−	−
2007	Evo48	3	+	−	−	3/3	−	−
	Evo49	3	+	+	−	2/3	−	1/3
	Evo51	4	+	+	3/4	4/4	1/4	−
	Evo52	4	+	−	−	4/4	−	−
	Evo53	3	+	−	−	3/3	−	−
	Evo54	3	+	−	−	3/3	−	−
Total	26	80	26	14	43/46	78/80	3/80	2/80

Of the three unassigned nestlings, maternity for one could not be assigned with >80% confidence and it was from a brood of four nestlings. Since the father for the maternally unassigned nestling was confirmed in the later paternity analysis, additional maternity analysis with known father did not assign it any genetic mother with >80% confidence either. In both analyses with unknown and known fathers, the nestling could not be assigned to any maternity, neither the putative mother, nor to any other candidate adult females in the population. The putative mother/offspring pair, identified as having more than two genotype mismatches, were characterized by negative LOD scores. Thus, the maternity of the nestling could not be resolved and the true genetic mothers were unlikely to have been sampled as all candidate females were excluded at least on basis of mismatches in two loci. These results also do not exclude the possibility that the offspring may have resulted from extrapair fertilization.

Next, we attempted to assign paternity for the 46 offspring from 14 complete families including the offspring with unassigned maternity. In the assignment analyses, the putative father was not successfully assigned for two of 46 offspring in two of 12 broods, while the remaining offspring could be assigned to a candidate male with >95% confidence (Table 3). For the 12 families (34 nestlings) from which the putative fathers alone had been sampled, putative fathers were assigned to all offspring with a >80% confidence. Thus, the putative father was excluded for two (2.5%) of 80 nestlings in two (7.7%) of 26 broods. When all other potential candidate adult males were tested against the two extra-pair chicks with known mothers, the true genetic father for one chick was detected with >95% confidence. However, no male emerged as a likely candidate father for the other nestling - none met even an 80% confidence criterion.

Of the nestlings, two were found to be unassigned to any maternity or paternity. Genetic sampling of adult males and females at the study area was not complete in the years, and we suspect — by analogy with other published studies (e.g. [22], [23]) - that the genetic parents of the two extra-pair nestlings were resident, unsampled territorial adults.

## Discussion

The main aim of this study was to gain insight into the mating system of the three-toed woodpeckers with the aid of rigorous statistical analyses (viz. the extremely high cumulative exclusion probability, distinct mismatch between social parents and extrapair offspring and the powerful likelihood-based approach) of genetic data. The results provide the first genetic evidence for the co-occurrence of polyandry and polygamy in the three-toed woodpecker. To this end, they add to our understanding of breeding behaviour of non-passerine birds, and to an increasing number of studies reporting occurrence of extra-pair fertilizations in natural bird populations (see [2], [4]).

### Extrapair Paternity of Three-Toed Woodpeckers

This is the first genetic study showing that EPP and QP occur within a single woodpecker species. Overall, however, this species is predominantly genetically monogamous. Since mate switching within a breeding season has never been visually observed in this species (see [5], [7]), EPPs and QPs predominantly result from extrapair copulations. Mate switching can, however, occur for example when male or female dies accidentally during the early breeding season.

The frequency of extrapair paternity varies markedly within and between species (see [2]). Our point estimate of the proportion of EPP is 2.5% which is much less than the average of *ca*. 11% in passerines, but more frequent than in some other genetically monogamous species such as the New Zealand saddlebacks *Philesturnus carunculatus* and robins *Petroica australis* where no EPP has ever been detected [24]. Comparative studies suggest that many factors such as phylogenetic history, breeding synchrony and breeding density, demands for paternal care, the rate of adult mortality as well as the intensity of sexual conflicts all influence the costs and benefits of extrapair copulations, and therefore, contribute to the variation in EPC frequency among species (see [2]). In the context of this study the question becomes: what might keep extra-pair fertilization rates low in three-toed woodpeckers as compared to the average extrapair paternity rate of *ca.* 11% for e.g. passerines [2]? We predict that the greatest potential of the need for paternal care hypothesis will be in explaining the differences in the level of EPP among the species because the high male investment in brood care is essential for female reproductive success. In three-toed woodpeckers, males allocate significantly more time to territory defence, cavity excavation and feeding young than females [5]. Nocturnal incubation and brooding as well as nest construction also constrain males with respect to social polygamy (e.g. [6]). In addition, since there are significant sex differences in the provision of various types of care and the total duration of different components of care, these differences could be another possible behavioural explanation [25] for the low extrapair paternity observed here.

Intraspecific variation in the frequency of EPP can occur at both at the spatial (e.g. the house sparrow *Passer domesticus*, [26]) and temporal levels (e.g. the red-winged blackbird *Agelaius phoeniceus*, [27]). Recent studies of extrapair paternity found a somewhat but not significantly (Fisher's exact test; *P* = 0.37) higher rate of EPP (3.6−5.5%) in a German population of three-toed woodpeckers [5], [7]. Although ecological factors could explain different levels of EPP in three-toed woodpeckers observed in this and earlier studies [5], [7], some additional potential explanations may be evoked. Firstly, the earlier studies may have lower statistical power due to the smaller sample size (*n* = 55 chicks, 95% CI: 41.79−63.21), the lower-resolution molecular tools (multi-locus DNA fingerprinting) and statistical methods (exclusion-based analysis) employed (see [28]). Secondly, opportunities to adopt alternative reproductive strategies may differ between populations (see [29]), for instance due to habitat differences between the German and Finnish populations: study area of the German population is 600−2700 meters above sea level [5], while the average altitude of the study area of the Finnish population is *ca*. 130 meters [5], [7], [10]. Thirdly, spatiotemporal fluctuations in population density and resources are likely to induce temporal variation in EPP frequency. However, further studies are needed to indentify proximate and ultimate determinants of EPP occurrence in the species.

### Quasi-Parasitism of Three-Toed Woodpeckers

We found a low (3.8%) frequency of extrapair maternity resulting from quasi-parasitism. This has rarely been reported in the related woodpeckers such as the lesser spotted woodpecker *Dendrocopos minor* [30]. A number of explanations have been put forward to explain the occurrence of QP [31]. The ‘female-driven QP’ suggests that a female may choose, or assent after an approach, to copulate with an extrapair male and goes on to lay one or more of her eggs in his nest. This option implies that females select ‘high quality’ males to fertilize their eggs, and either avoid the costs of parental care associated with provisioning some young or benefit from the chosen males’ ‘good genes’ or directly from behavioural or other contributions such as territory quality (e.g. [32]). Another one of the main hypothesis suggests that QP in non-passerine birds is an insurance mechanism against the potential detrimental effect of inbreeding, or more simply, males' own low quality mate [31]. Nevertheless, given the low level of QP in this study population it seems unlikely that QP is an inbreeding avoidance strategy as high levels of extrapair copulations would be expected in such a case (cf. [33]). Furthermore, our data is thin about the actual relatedness between partners, making it difficult to test the inbreeding avoidance hypothesis with much confidence.

Interestingly, the rate of QP for the females in three-toed woodpeckers (3.75%) is at the lower range of estimates reported for many shorebirds (e.g. Common sandpiper. *Actitis hypoleucos*, 5.7%, [1]) and passerines (e.g. Sand martin *Riparia riparia*, 2.4%, [31]). The limits for QP could arise from the species characteristics such as a high degree of male parental care, long duration of cavity excavation (and thus a narrow time frame for fertilization), long day-time incubation and brooding shifts (more than 3 hours, [34]), and few re-mating opportunities [5], all of which are likely to constrain both males and females in their ability to obtain additional mates, and also limit their ability to seek extrapair partners. However, the estimates of both EPP and QP obtained here should be considered with caution. One potential caveat is that we did not sample unhatched eggs or dead chicks. This affects the estimates by making them conservative under the assumption that mortality before sampling is random in respect to EPP and QP. Another possible bias in the estimates comes from the idea that nests with QP may be more heavily predated if females defend them less vigorously. It is also worth noting that since the estimated rates of EPP and QP are just based on relatively few nestlings sampled, the confidence limits of these estimates are probably broad and hence the estimates are potentially imprecise.

We detected no case of conspecific brood parasitism (CBP) due to egg dumping in this study while a single case of CBP, as a result of egg-dumping or QP, has been reported from a German population of three-toed woodpeckers [5]. This suggests that the CBP stemming from egg dumping must be rare in our study population. Overall, the two populations did not differ significantly in the frequency of extrapair offspring (Fisher's exact test; *P* = 0.41) or proportion of broods containing one or more extrapair young (Fisher's exact test; *P* = 0.61).

In conclusion, our results of genetic analysis found the co-occurrence of low levels of EPP and QP in the three-toed woodpeckers. Although alternative explanations may exist for the observations in our study species (see [2], [4]), our data are consistent with the hypothesis that a high degree of male parental care play an important role in explaining low rates of EPP and QP across species. The information provided in this study further allows us to examine the success of male and female mating patterns, as well as to understand the evolutionary significance and life-history correlates of promiscuity in birds.
